# A New Sail-Backed Styracosternan (Dinosauria: Ornithopoda) from the Early Cretaceous of Morella, Spain

**DOI:** 10.1371/journal.pone.0144167

**Published:** 2015-12-16

**Authors:** José Miguel Gasulla, Fernando Escaso, Iván Narváez, Francisco Ortega, José Luis Sanz

**Affiliations:** 1 Unidad de Paleontología, Universidad Autónoma de Madrid, Cantoblanco, Madrid, Spain; 2 Grupo de Biología Evolutiva, Universidad Nacional de Educación a Distancia, Madrid, Spain; College of the Holy Cross, UNITED STATES

## Abstract

A new styracosternan ornithopod genus and species is here described based on a partial postcranial skeleton and an associated dentary tooth of a single specimen from the Arcillas de Morella Formation (Early Cretaceous, late Barremian) at the Morella locality, (Castellón, Spain). *Morelladon beltrani* gen. et sp. nov. is diagnosed by eight autapomorphic features. The set of autapomorphies includes: very elongated and vertical neural spines of the dorsal vertebrae, midline keel on ventral surface of the second to fourth sacral vertebrae restricted to the anterior half of the centrum, a posterodorsally inclined medial ridge on the postacetabular process of the ilium that meets its dorsal margin and distal end of the straight ischial shaft laterally expanded, among others. Phylogenetic analyses reveal that the new Iberian form is more closely related to its synchronic and sympatric contemporary European taxa *Iguanodon bernissartensis* and *Mantellisaurus atherfieldensis*, known from Western Europe, than to other Early Cretaceous Iberian styracosternans (*Delapparentia turolensis* and *Proa valdearinnoensis*). The recognition of *Morelladon beltrani* gen. et sp. nov. indicates that the Iberian Peninsula was home to a highly diverse medium to large bodied styracosternan assemblage during the Early Cretaceous.

## Introduction

Until recently, the Early Cretaceous European fossil record of styracosternan iguanodonts was composed of basal representatives of the node-based clade Hadrosauriformes (*sensu* Sereno [1]), or members of the outgroup. *Delapparentia*, *Hypselospinus*, *Iguanodon*, *Mantellisaurus* and *Proa* were the hadrosauriform styracosternans recognized from several Lower Cretaceous formations [2–5]. A recent re-evaluation of the phylogenetic relationships indicates a new subclade of non-hadrosauriform styracosternans (‘iguanodontoids’) and includes most of these previously considered European hadrosauriforms. In this new view, all of the European Cretaceous large-bodied styracosternans lie outside of the Hadrosauriformes according to a new definition of the clade (see [6]).

Presently, the Lower Cretaceous Iberian styracosternan iguanodontians species are the lower Barremian *Delapparentia turolensis* [2] and *Iguanodon galvensis* [5], the upper Barremian *Iguanodon bernissartensis* and *Mantellisaurus atherfieldensis* [7–10], and the lower Albian *Proa valdearinnoensis* [3]. However, it should be noted that Norman [6] considers *Delapparentia turolensis* provisionally as a *nomem dubium*.

**Fig 1:**
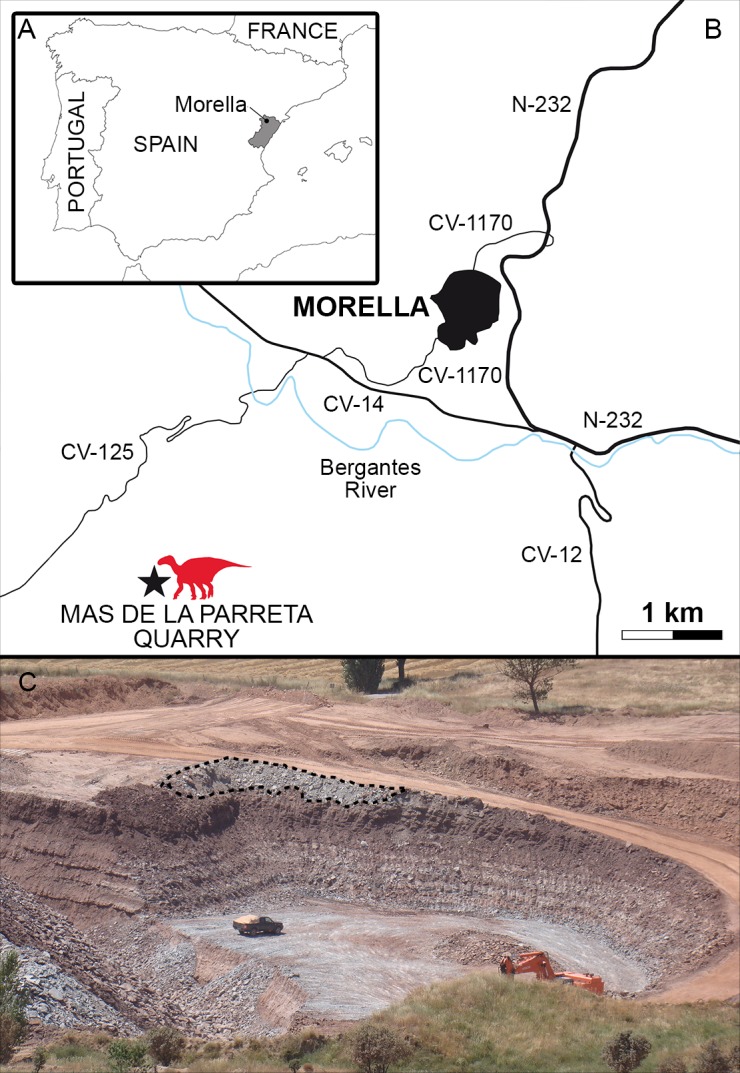
Geographic location of the holotype specimen of *Morelladon beltrani* (CMP-MS-03). (A) Castellón province and Morella locality in Spain. (B) Schematic regional map of area of Morella locality indicating location of the Mas de la Parreta Quarry (black star). (C) Photography showing the location of the holotype site in the CMP-MS area (dash line).

Here, a new specimen is described from the Morella locality (Castellón province, Spain) (Fig 1). It comprises a well-preserved partial skeleton of a medium-sized iguanodontian, consisting of a complete right dentary tooth, six almost complete dorsal vertebrae, a dorsal centrum, several fragments of dorsal neural spines, two dorsal ribs fragments, a nearly complete sacrum, two haemal arches, ilia, incomplete pubes and ischia, and the right tibia. The specimen was found in a body of red clays belonging to the upper Barremian Arcillas de Morella Formation [11]. Here we provide a detailed description of this new taxon and discuss its phylogenetic relationships within Iguanodontia.

Institutional Abbreviations: CEUM, College of Eastern Utah Prehistoric Museum, Price, USA; CMP-MS, Mas de la Parreta Quarry-Mas de Sabater, Morella, Spain; GPIT, Geologisches und Palaontologisches Institut der Universität Tübingen, currently the Institut für Geowissenschaften, Tübingen, Germany IRSNB, Institut royal des Sciences naturelles de Belgique, Brussels, Belgium; MNHN, Muséum national d’Histoire naturelle, Paris, France; MPZ, Museo de Ciencias Naturales de la Universidad de Zaragoza, Zaragoza, Spain. NHMUK, The Natural History Museum, London, UK; YHZ, Yizhou Fossil Museum, Yixian, Peoples Republic of China.

## Materials and Methods

### Paleontological Ethics Statements

All necessary permits were obtained for the described study, which complied with all relevant regulations. The holotype specimen was collected under permits obtained from the Dirección General de Cultura [Consellería de Educación, Cultura y Deporte-Generalitat Valenciana (2013/0016Cs)] for work conducted in the Mas de la Parreta Quarry-Mas de Sabater.

The holotype specimen (CMP-MS-03) of *Morelladon beltrani* gen et sp nov. described in this paper is housed at the Museo de la Valltorta (Tirig, Castellón), part of the Museums Survey of the Generalitat Valenciana.

### Nomenclatural Acts

The electronic edition of this article conforms to the requirements of the amended International Code of Zoological Nomenclature, and hence the new names contained herein are available under that Code from the electronic edition of this article. This published work and the nomenclatural acts it contains have been registered in ZooBank, the online registration system for the ICZN. The ZooBank LSIDs (Life Science Identifiers) can be resolved and the associated information viewed through any standard web browser by appending the LSID to the prefix "http://zoobank.org/". The LSID for this publication is: urn:lsid:zoobank.org:pub:E39BF4C9-831F-459D-B238-CD7FC7927F5D. The electronic edition of this work was published in a journal with an ISSN, and has been archived and is available from the following digital repositories: PubMed Central, LOCKSS.

## Results and Discussion

### Systematic palaeontology

Dinosauria Owen, 1842 [12]

Ornithischia Seeley, 1887 [13]

Ornithopoda Marsh, 1881 [14]

Iguanodontia Dollo, 1888 [15] *sensu* Norman, 2015 [6]

Ankylopollexia Sereno, 1986 [16] *sensu* Norman, 2015 [6]

Styracosterna Sereno, 1986 [16] *sensu* Norman, 2015 [6]

*Morelladon* gen nov.

urn:lsid:zoobank.org:act:2BEA6D12-8836-4F4C-9616-134FDDC9618E

*Morelladon beltrani* sp nov.

urn:lsid:zoobank.org:act:EEB70705-B246-48D8-8B79-C5E0A8C6C38C

#### Etymology

The generic name is derived from *Morella* (the name of the type locality) and *odon* (Greek word for “tooth”). The specific name is for Víctor Beltrán, for his involvement and collaboration in the localization of the different fossil sites at the Mas de la Parreta Quarry.

#### Holotype

CMP-MS-03, a partial skeleton including a complete right dentary tooth, six almost complete dorsal vertebrae, a dorsal centrum, several fragments of dorsals neural spines, two dorsal ribs fragments, a nearly complete sacrum, two haemal arches, ilia, incomplete pubes and ischia, and the right tibia (Figs 2–14). Measurements of select elements of *Morelladon beltrani* are given in S1 Supporting Information.

**Fig 2:**
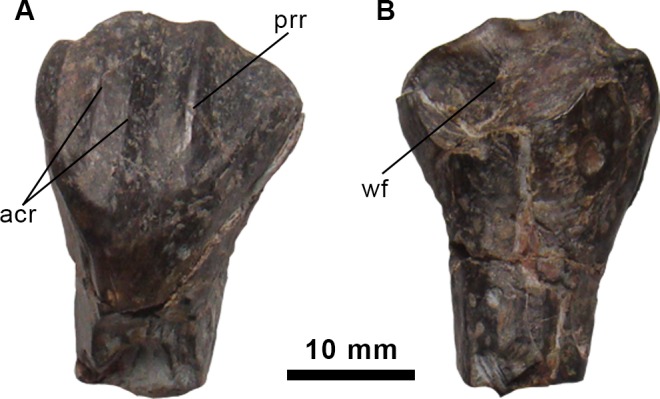
Dentary tooth of the holotype specimen of *Morelladon beltrani* (CMP-MS-03). Dentary tooth in medial or lingual (A) and lateral or labial (B) views. Abbreviations: acr, accessory ridge; prr, primary ridge; wf, wear facet.

**Fig 3:**
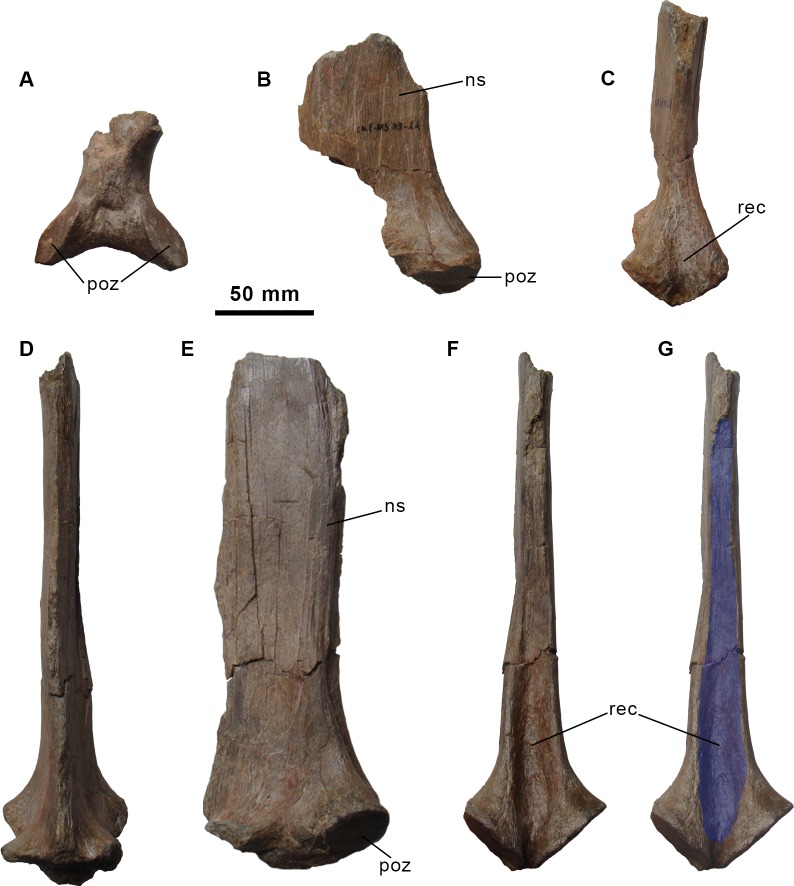
Partial dorsal neural arch and neural spines of the holotype specimen of *Morelladon beltrani* (CMP-MS-03). Partial anterior dorsal neural arch in ventral (A) view. Partial anterior dorsal neural spine in left lateral (B) and posterior (C) views. Partial posterior dorsal neural spine in anterior (D), left lateral (E) and posterior (F, G) views. Abbreviations: ns, neural spine; poz, postzygapophysis; rec, vertical recess.

**Fig 4:**
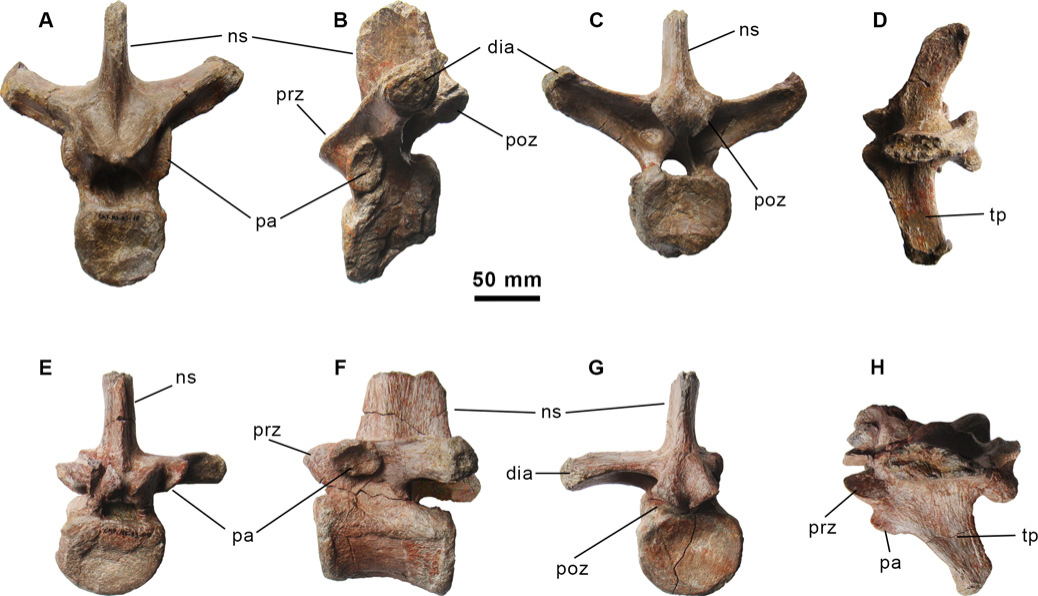
Dorsal vertebrae of the holotype specimen of *Morelladon beltrani* (CMP-MS-03). CMP-MS-03-10 in anterior (A), left lateral (B), posterior (C) and dorsal (D) views. CMP-MS-03-09 in anterior (E), left lateral (F), posterior (G) and dorsal (H) views. Abbreviations: dia, diapophysis; ns, neural spine; pa, parapophysis; poz, postzygapophysis; pre, prezygapophysis; tp, transverse process.

**Fig 5:**
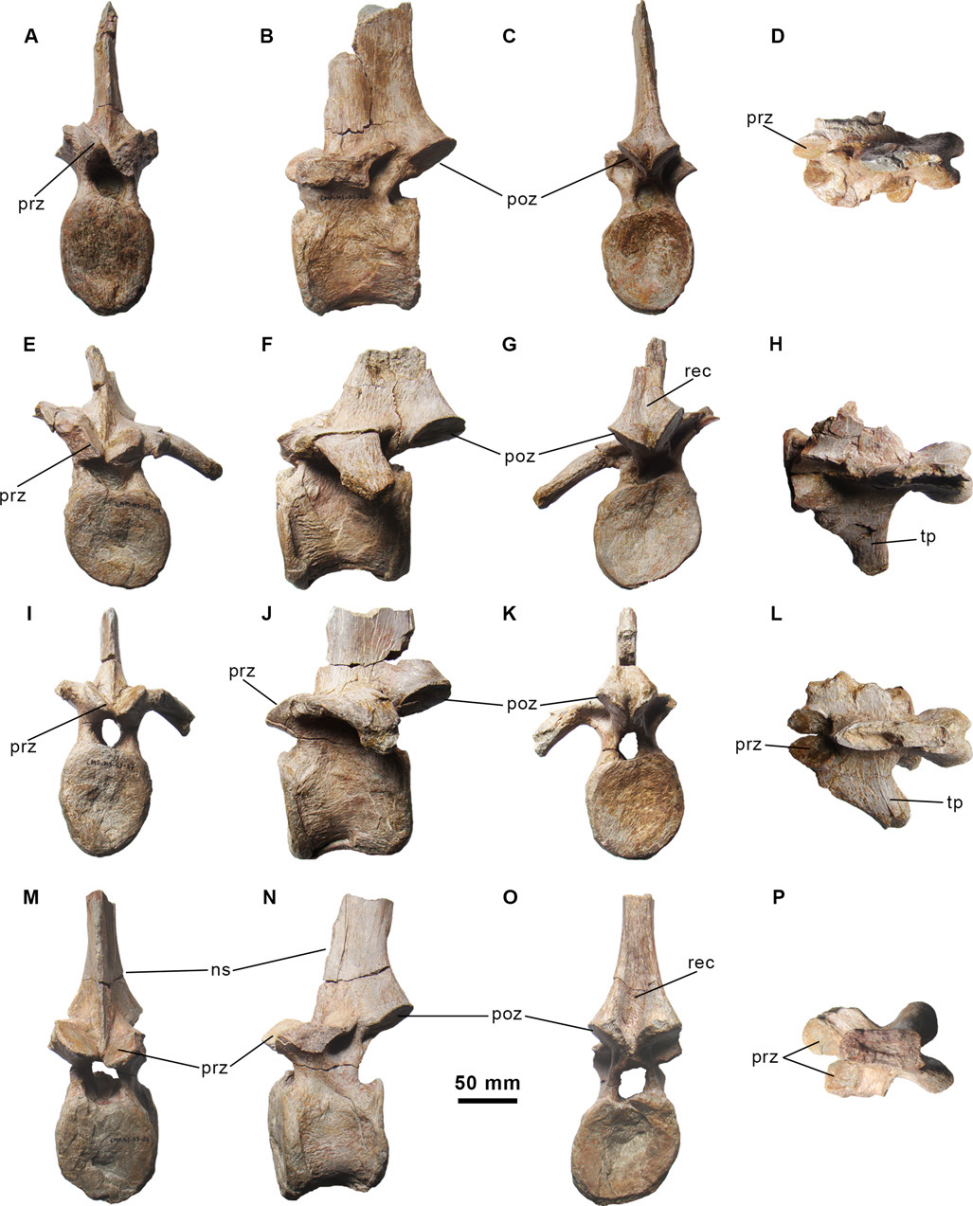
Dorsal vertebrae of the holotype specimen of *Morelladon beltrani* (CMP-MS-03). CMP-MS-03-06 in anterior (A), left lateral (B), posterior (C) and dorsal (D) views. CMP-MS-03-05 in anterior (E), left lateral (F), posterior (G) and dorsal (H) views. CMP-MS-03-07 in anterior (I), left lateral (J), posterior (K) and dorsal (L) views. CMP-MS-03-05 in anterior (M), left lateral (N), posterior (O) and dorsal (P) views. Abbreviations: ns, neural spine; poz, postzygapophysis; pre, prezygapophysis; rec, vertical recess; tp, transverse process.

**Fig 6:**
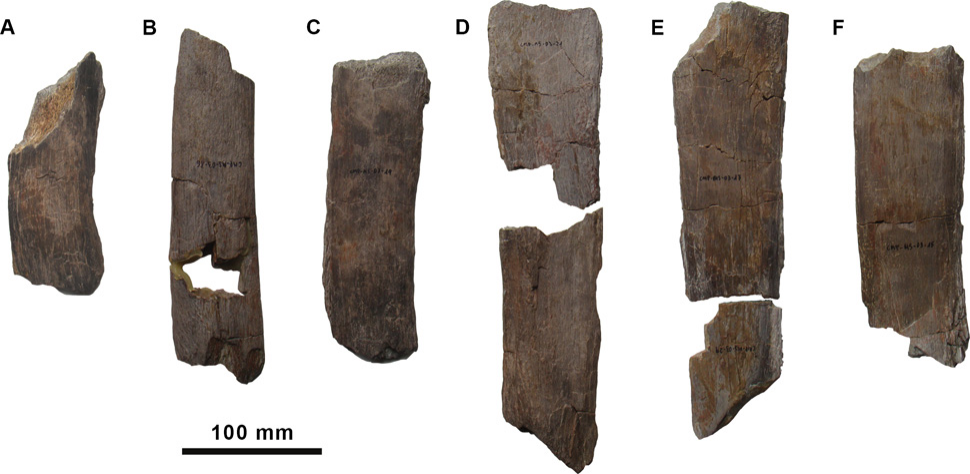
Partial dorsal neural spines of the holotype specimen of *Morelladon beltrani* (CMP-MS-03). CMP-MS-03-25 in left lateral (A) view. CMP-MS-03-16 in left lateral (B) view. CMP-MS-03-19 in left lateral (C) view. CMP-MS-03-21 in left lateral (D) view. CMP-MS-03-17 and -29 in left lateral (E) view. CMP-MS-03-15 in left lateral (F) view.

**Fig 7:**
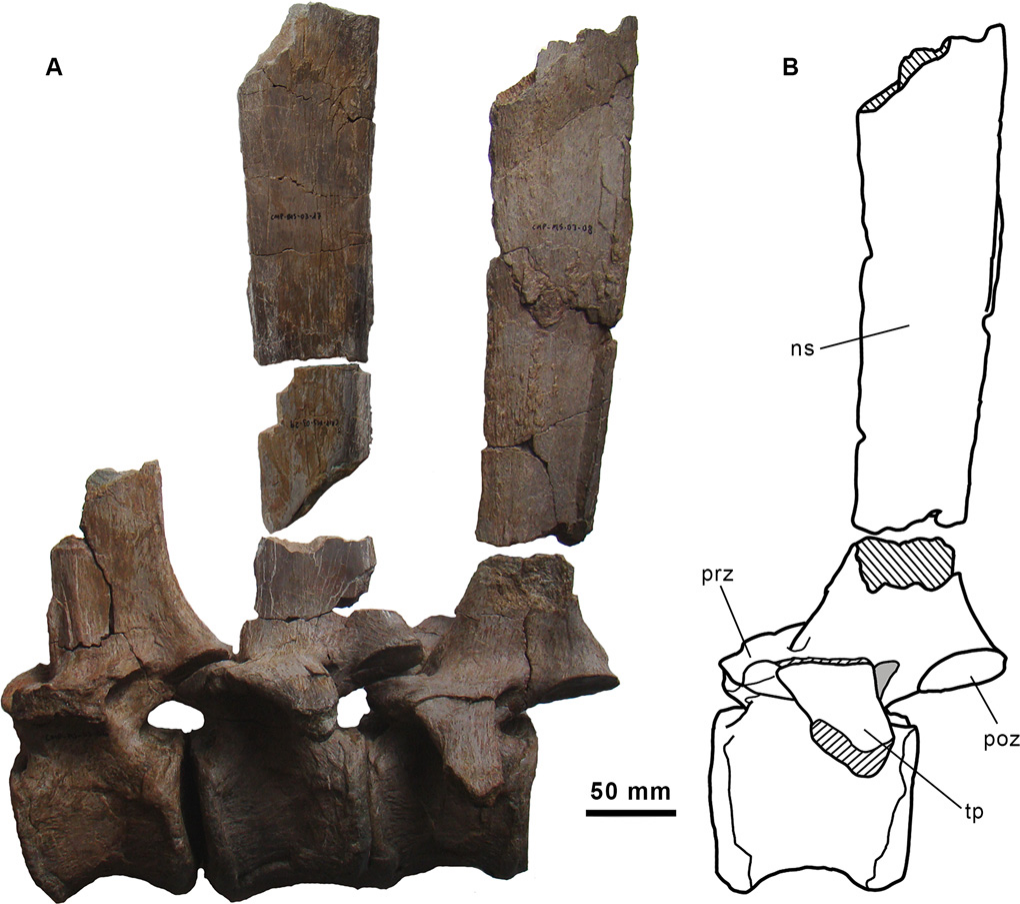
Dorsal vertebrae series of the holotype specimen of *Morelladon beltrani* (CMP-MS-03). CMP-MS-03-06, -07 (including CMP-MS-03-17 and -29) and -05 (including CMP-MS-03-08) in left lateral (A) view. Interpretive drawing of CMP-MS-03-05 (including CMP-MS-03-08 neural spine) in left lateral (B) view. Abbreviations: ns, neural spine; poz, postzygapophysis; pre, prezygapophysis; rec, vertical recess; tp, transverse process.

**Fig 8:**
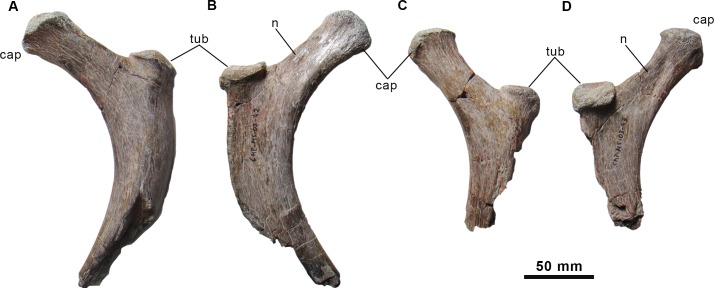
Partial dorsal ribs of the holotype specimen of *Morelladon beltrani* (CMP-MS-03). CMP-MS-03-42 in anterior (A) and posterior (B) views. CMP-MS-03-43 in anterior (C) and posterior (D) views. Abbreviations: cap, capitulum; n, neck; tub, tuberculum.

**Fig 9:**
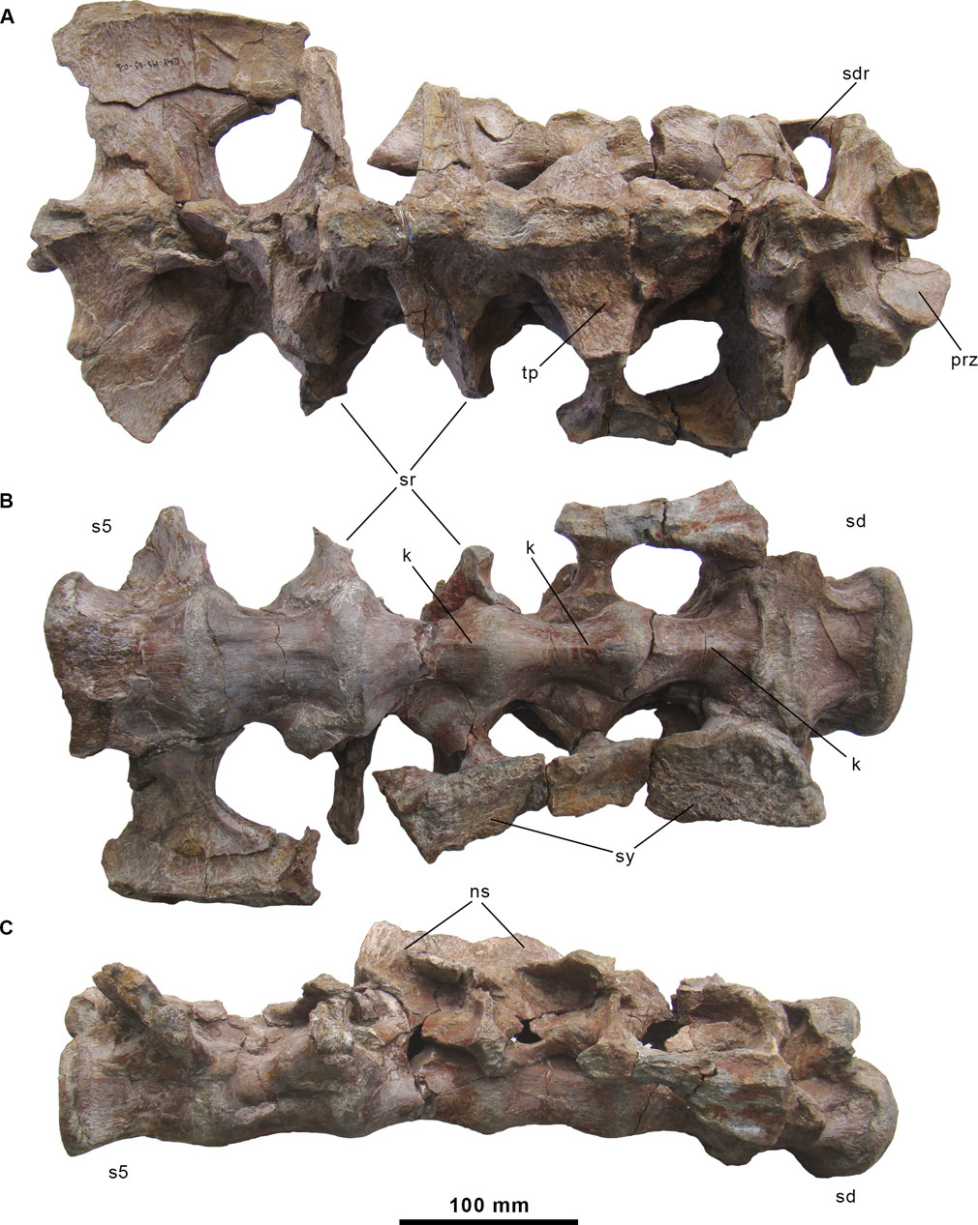
Sacrum of the holotype specimen of *Morelladon beltrani* (CMP-MS-03). Sacrum (CMP-MS-03-01) in dorsal (A), ventral (B) and right lateral (C) views. Abbreviations: k, ventral keel; ns, neural spine; pre, prezygapophysis; sd, sacrodorsal vertebra; sdr, sacrodorsal rib; sr, sacral ribs; sy, sacral yoke; s5, sacral vertebra 5; tp, transverse process.

**Fig 10:**
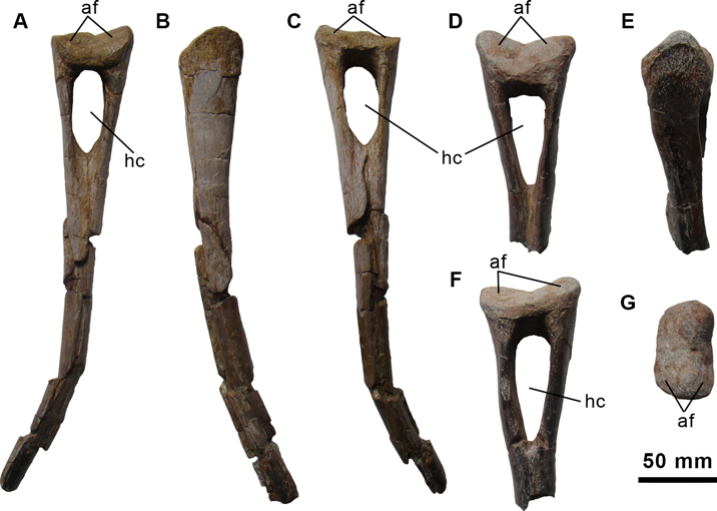
Haemal arches of the holotype specimen of *Morelladon beltrani* (CMP-MS-03). CMP-MS-03-14 in anterior (A), left lateral (B) and posterior (C) views. CMP-MS-03-13 in anterior (D), left lateral (F), posterior (C) and proximal (G) views. Abbreviations: af, articular fact; hc, haemal canal.

**Fig 11:**
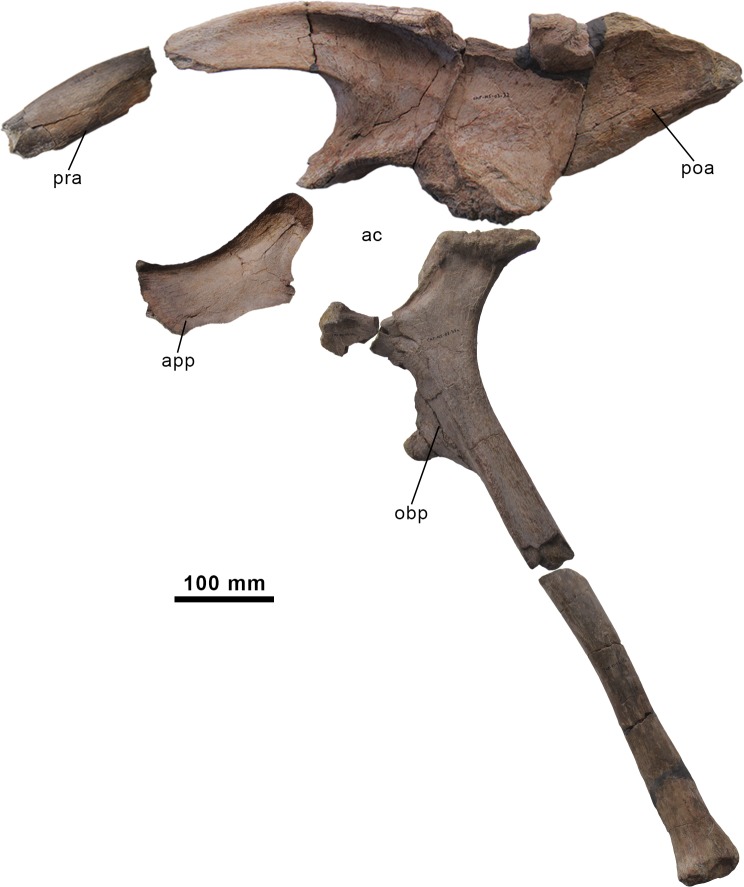
Pelvic girdle of the holotype specimen of *Morelladon beltrani* (CMP-MS-03). Left pelvic elements (ilium, pubis and ischium) in left lateral view. Abbreviations: ac, acetabulum; app, anterior pubic process; obp, obturator process; poa, postacetabular process; pra, preacetabular process.

**Fig 12:**
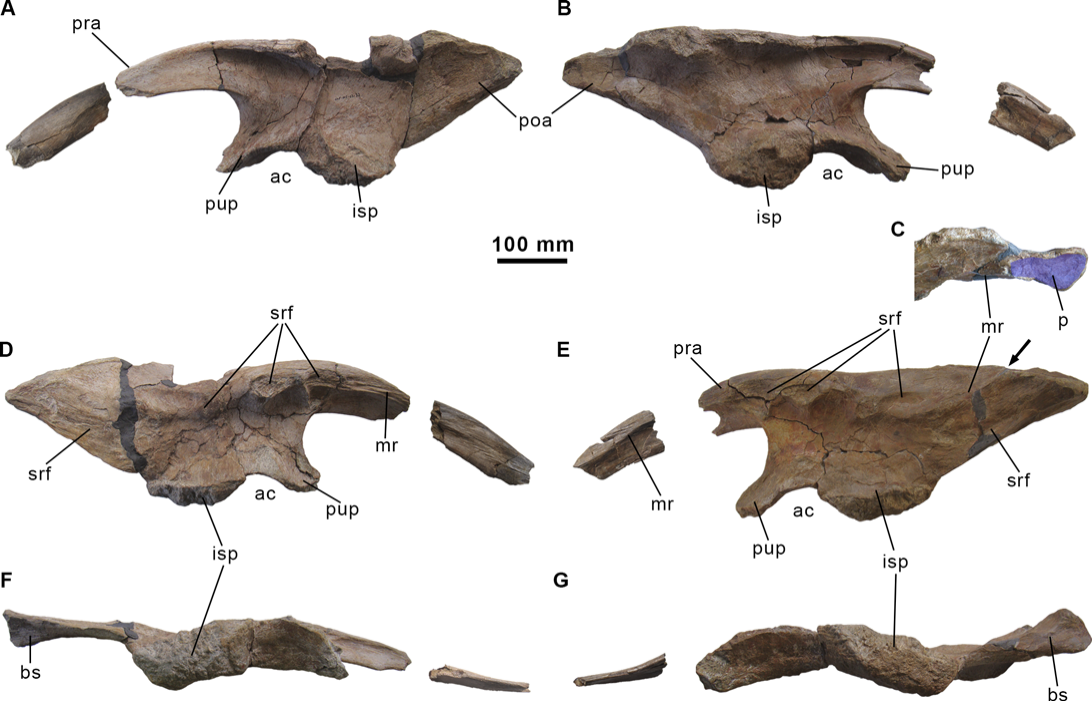
Ilia of the holotype specimen of *Morelladon beltrani* (CMP-MS-03). Left ilium (CMP-MS-03-32) in lateral (A), medial (C) and ventral (E) views. Right ilium (CMP-MS-03-31) in lateral (B), medial (D) and ventral (F) views. Abbreviations: ac, acetabulum; bs; brevis shelf; isp, ischiadic peduncle; mr, medial ridge; p, dorsal platform; poa, postacetabular process; pra, preacetabular process; pup, pubic peduncle; srf, sacral rib facets. Arrow in (E) indicates point in which the medial ridge meets the dorsal margin of the ilium.

**Fig 13:**
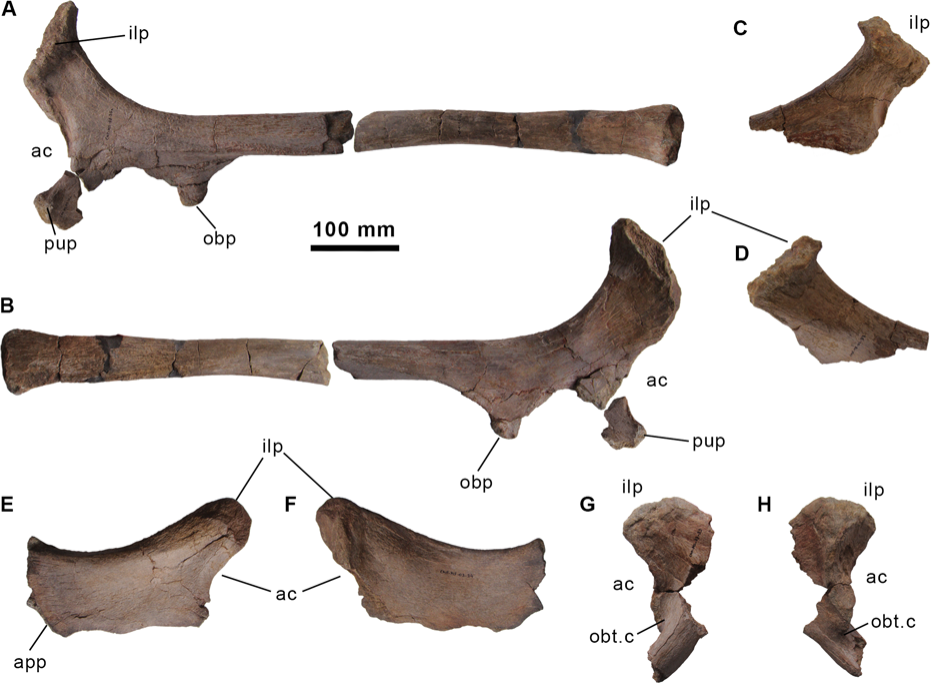
Ischia and pubes of the holotype specimen of *Morelladon beltrani* (CMP-MS-03). Left ischium (CMP-MS-03-35) in lateral (A) and medial (B) views. Right ischium (CMP-MS-03-37) in lateral (D) and medial (E) views. Right pubis (CMP-MS-03-34) in medial (E) and lateral (F) views. Left pubis (CMP-MS-03-33) in medial (G) and lateral (H) views. Abbreviations: ac, acetabulum; app, anterior pubic process; ilp, iliac peduncle; obp, obturator process; obt.c, obturator channel; pup, pubic peduncle.

**Fig 14:**
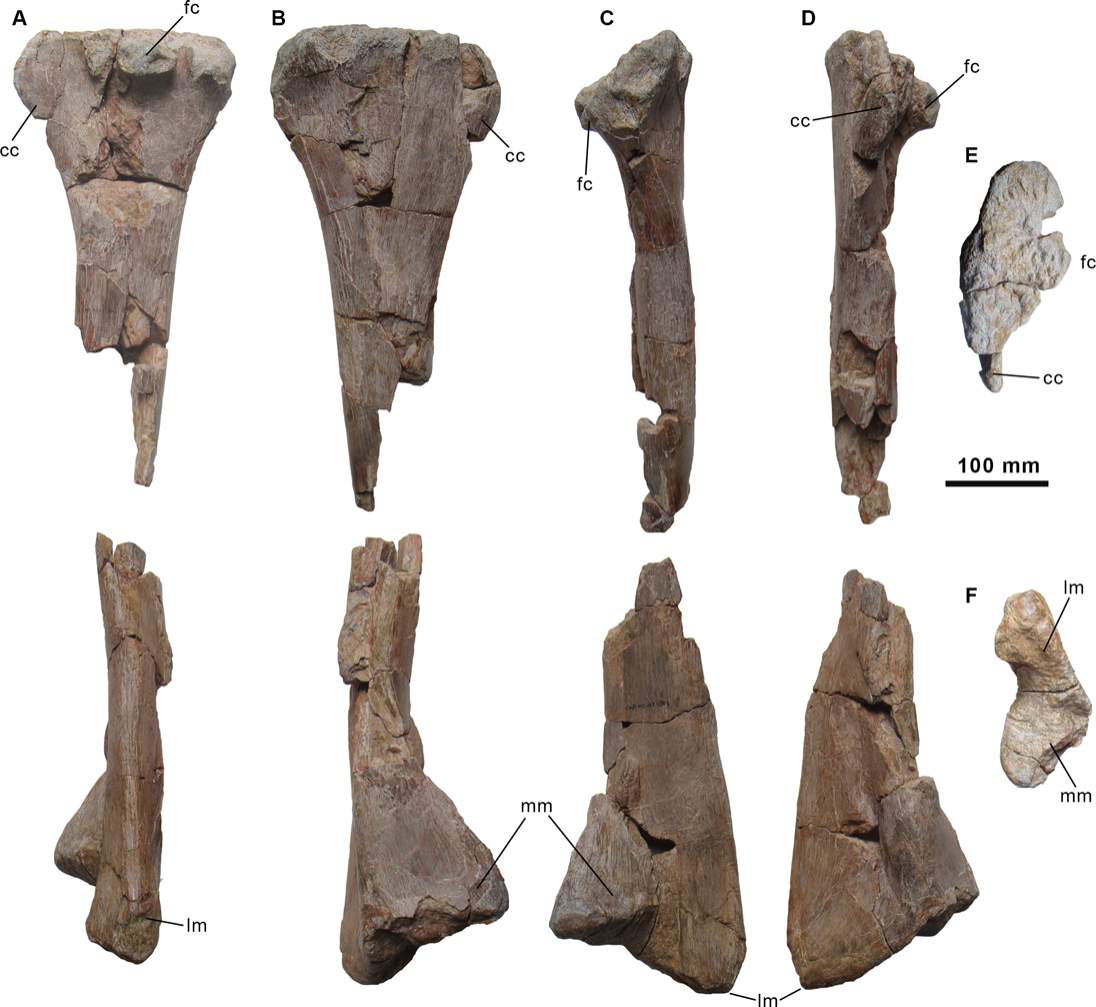
Tibia of the holotype specimen of *Morelladon beltrani* (CMP-MS-03). Left tibia (CMP-MS-03-30) in lateral (A), medial (B), posterior (C), anterior (D), proximal (E) and distal (E) views. Abbreviations: cc, cnemial crest; fc, fibular condyle; lm, lateral malleolus; mm, medial malleolus.

#### Specific diagnosis

A styracosternan iguanodontian with the following autapomorphies: dorsal neural spines are at least 4.3 times centrum height, extremely tall and deep vertical recess between postzygapophyses and proximal portion of the neural spines in mid-posterior dorsal vertebrae (2.6 times spine anteroposterior length), dorsosacral rib distally fused to the sacral yoke, midline ventral keel restricted to the anterior half of the centrum in sacrals 2 and 3, slightly pronounced and broad ventral keel restricted to the anterior half of the centrum in sacral 4, sacrodorsal and first sacral rib facets closely spaced and located dorsal to the remaining sacral rib facets near the dorsal margin of the ilium, medial ridge on the postacetabular process slopes posterodorsally to meet the point in which the dorsal margin of the ilium slopes posteroventrally forming a medially expanded and shallowly concave dorsal platform at posterior portion of the postacetabular process, and distal end of the ischium with a D cross-section due to lateral expansion.

#### Locality and horizon

The specimen was collected in the Mas de Sabaté (CMP-MS) site within the Mas de la Parreta Quarry at Morella, Spain (Fig 1). The Arcillas de Morella Formation has traditionally been dated as early Aptian in age [17], but new palynological data indicates a Barremian age for this Iberian Formation [11].

### Description and comparisons

#### Dentition

Fourteen teeth were recovered, but except CMP-MS-03-89 all the elements are poorly preserved and consist of roots and portions of the crown bases that do not allow a detailed description. CMP-MS-03-89 (Fig 2) is a right dentary tooth that preserves the proximal portion of the root and the basal half of the crown. The general aspect of this tooth is similar to that seen in ankylopollexian ornithopods. The crown is heavily worn and the marginal denticles on the mesial and distal margins are not preserved. The crown is labiolingually narrow and mesiodistally expanded. The lingual surface of the crown is enamelled and bears a prominent distally offset primary ridge. Two narrow, subparallel accessory ridges are located mesial to the primary ridge (Fig 2A). The labial surface of the crown possesses an almost vertical, slightly concave wear facet (Fig 2B).

#### Dorsal vertebrae

Six almost complete dorsal vertebrae (CMP-MS-03, -04, -05, -06, -07, -09, and -10), a posterior dorsal centrum (CMP-MS-03-02) and several fragments of dorsal neural spines are preserved (Figs 3–7). The identification of the position of each dorsal vertebra is based on the assumption of a sequence of 16 dorsal vertebrae as in *Iguanodon bernissartensis* [18] and *Mantellisaurus atherfieldensis* [19].

A fragmentary neural arch probably represents a second or third dorsal vertebra based on the shape and orientation of the postzygapophyses (Fig 3A). The facets of the postzygapophyses are ventrolaterally directed forming an angle higher than 90 degrees between them and are relatively small, flat and oval in profile.

The most anterior of the preserved dorsal vertebrae is approximately the fifth (CMP-MS-03-10) (Fig 4A–4D). The centrum is distorted and exhibits an unnatural inclination, so the anterior articular facet is located ventral to the posterior articular facet when it is viewed laterally (Fig 4B). The anterior articular facet is flat to gently concave whereas the posterior articular facet is concave. Both articular facets are almost circular with the dorsal rim related to the floor of the neural canal nearly straight. Anteriorly, the neural canal is large and circular whereas it is smaller and subtriangular posteriorly. The parapophyses are large, oval and gently concave (Fig 4B). The parapophyses are located on the lateral surface of the neural arch, between the prezygapophyses and the bases of the transverse processes. The robust transverse processes are triangular in cross-section with the apex pointing ventrally and dorsolaterally directed (Fig 4A and 4C). The transverse processes terminate in thickened and rugose diapophyses. Posteriorly, the bases of the transverse processes are deeply excavated. The steeply inclined prezygapophyses are extended beyond the anterior margin of the centrum and separated from the midline by a groove. The postzygapophyses are located at the base of the neural spine and separated by a shallow sulcus (Fig 4C). The smooth articular facets of the postzygapophyses are oval and ventrolaterally directed. The postzygapophyses extend beyond the posterior margin of the centrum. Although it is broken, the neural spine is anteroposteriorly expanded and transversely thicker anteriorly than posteriorly. The anterior and posterior margins of the neural spine are sharp (Fig 4A and 4C).

A fragmentary base of a dorsal neural spine (CMP-MS-03-11) that preserves both postzygapophyses is probably a remnant of a sixth or seventh dorsal (Fig 3B and 3C). The morphology of the neural spine is similar to those of CMP-MS-03-09 and CMP-MS-03-10. The facets of the postzygapophyses form an intermediate between the aforementioned vertebrae.

Dorsal vertebra CMP-MS-03-09 (Fig 4E–4H) is approximately the eighth, based on its size, the position of the parapophyses and the morphology of the transverse processes. As in the fifth dorsal vertebra, the centrum exhibits an unnatural inclination, so the anterior articular facet is located dorsal to the posterior articular facet in lateral view (Fig 4F). The anterior articular facet of the centrum is flat to slightly concave whereas the posterior articular facet is concave. Both oval articular facets are broader transversely than they are deep dorsoventrally with expanded rims. In lateral view the centrum is low relative to the most posterior centra, cylindrical and ventrally has a narrow keel. The neural arch is similar to that seen in the fifth dorsal. However, in this vertebra the parapophyses are located more dorsally on a short projection between the prezygapophyses and the bases of the transverse processes (Fig 4E). The parapophyses are large, oval, gently concave, and ventromedially inclined. Only the left transverse process is preserved and horizontal (Fig 4G). It is triangular in cross-section with the apex pointing ventrally and posterolaterally directed (Fig 4H). The transverse processes terminate in thickened and rugose diapophyses. Posteriorly the base of the transverse process is deeply excavated. Although the right prezygapophysis is crushed, both are steeply inclined and separated from the midline by a groove. The prezygapophyses are slightly extended beyond the anterior margin of the centrum. The postzygapophyses are located at the base of the neural spine and separated by a shallow sulcus (Fig 4G). A shallow vertical recess between both postzygapophyses is located dorsal to the sulcus. Its facets are ventrolaterally directed forming an angle higher than 90 degrees between them. The postzygapophyses extended beyond the posterior margin of the centrum. The proximal portion of the neural spine is preserved and is anteroposteriorly expanded. The lateral sides of the neural spine are thickened being more developed the mid-anterior portion. Both the anterior and posterior margins of the neural spine are sharp (Fig 4E and 4G).

The remaining vertebrae are approximately the eleventh, twelfth and thirteenth dorsals (Fig 5A–5L). These vertebrae have amphicoelous centra in which the posterior articular facets are deeply concave. The anterior and posterior articular facets of the centra are oval in profile. The centra are spool-shaped with expanded and thickened anterior and posterior articular margins in ventral view, but form rectangular cylinder when viewed laterally (Fig 5B, 5F and 5J). A prominent ventral keel is present in the eleventh and twelfth dorsals. The prezygapophyses are slightly extended beyond the anterior margin of the centra and increase in size through the series. Their oval facets are ventrolaterally directed forming an angle of 90 degrees between them. The ventral margins of the prezygapophyes are nearly in contact. The parapophyses, restricted to small and rugose facets, are located on the anterior margins of the transverse processes near their bases (Fig 5A, 5E and 5I). Only the left transverse processes of the twelfth and thirteen dorsals are nearly complete but distorted (Fig 5A and 5E). Despite distortion, it appears that the transverse processes were probably horizontal. In dorsal view, the left transverse process of the twelfth dorsal is posterolaterally directed (Fig 5L), whereas in the preceding dorsal it is laterally directed. In these vertebrae the posterior surface of the base of the transverse processes are less excavated than the more anterior dorsals. The postzygapophyses are located at the base of the neural spine level with the posterodorsal margin of the transverse processes and they project slightly ventrolaterally. A large and deep vertical recess at the base of the neural spine separates the postzygapophyses (Fig 5C, 5G and 5K). The facets of the postzygapophyses are oval and ventrolaterally directed forming an angle of 90 degrees between them. The prezygapophyses are extended well beyond the posterior margin of the centra and increase in size through the series. The neural spines are anteroposteriorly expanded and transversely flattened. The anterior margins of the neural spines are sharp-edges as is the case for the posterior margin of the eleventh dorsal (Fig 5A and 5C). In the other two succeeding dorsals only a portion of the neural spine immediately above the postzygapophyses is preserved. Except in the thirteenth, the anterior half of the neural spine in the preceding dorsals is mediolaterally thickened. However, in the thirteenth dorsal the posterior margin of the neural spine is somewhat thicker.

A nearly complete dorsal vertebra (Fig 5M–5P), a dorsal centrum and a neural spine with postzygapophyses (Fig 3D–3G) represent elements of the posterior dorsal series. These elements are different from those of the anterior and mid-dorsal series. Both centra are anteroposteriorly compressed, compared with the preserved anterior and mid-dorsal vertebrae, with thickened and transversely expanded rims. The anterior articular facet is flat whereas the posterior articular facet is deeply concave. The transverse processes are broken showing a rhomboid cross-section. The prezygapophyses are ventromedially directed and separated from the midline by a shallow groove. The postzygapophyses are located at the base of the neural spine and separated by an extremely tall and deep recess that extends apically along the preserved neural spine (Fig 3F, 3G and 5O). The smooth articular facets of the postzygapophyses are ventromedially directed and oval. The postzygapophyses extend beyond the posterior margin of the centrum. The neural spines are anteroposteriorly shortened with sharp anterior margins and thickened posterior margins (Fig 3D, 3F, 5M and 5O). The borders that define the posterior recess on the neural spines are sharp.

Several incomplete dorsal neural spines are also preserved (Fig 6). The three most complete (CMP-MS-03-17, -16, -18) are 310, 240 and 235 mm in height, respectively. The morphology and texture of the surface of the neural spine fragment CMP-MS-03-08 is similar to that of CMP-MS-03-05 (thirteenth dorsal). Similarly, the neural spine fragments CMP-MS-03-17 and CMP-MS-03-29 are probably related with the twelfth of the series (CMP-MS-03-07) of *Morelladon*. This implies, at least, for two mid-dorsals that neural spines height are more than 4.3 times the height of its respective centra (Fig 7).

The morphology of the dorsal vertebrae of *Morelladon* resembles those of *Mantellisaurus atherfieldensis* [4,19] in having relatively rectangular cylindrical centra. As in *Hypselospinus fittoni* [6] and *Mantellisaurus atherfieldensis* [4,19] dorsal vertebrae present a ventral midline keel, however, the ventral keel disappears in the twelfth dorsal in *Jinzhousaurus yangi* [20]. The absence of thickened articular margin in the dorsal centra distinguishes *Morelladon* from *Hypselospinus fittoni*. As is widely distributed among styracosternan iguanodontians, a deep recess between postzygapophyses is present in mid-posterior dorsal of *Morelladon*. However, the vertical recess of *Morelladon* is unique among styracosternans in being remarkably tall, (as much as 2.6 times spine axial length, compared to 0.9 in *Iguanodon bernissartensis* (“Individu S”) [6], 1 in *Barilium dawsoni* (NHMUK R798) [21] or 0.7 in *Eolambia* (CEUM 52053). The dorsal neural spines of *Morelladon* are remarkably elongated distinguishing from those of *Barilium dawsoni* [21], *Bolong yixianensis* [22], *Hippodraco scutodens* [23], *Iguanacolossus fortis* [23], *Iguanodon bernissartensis* [4,18], *Jinzhousaurus yangi* [20], *Lanzhousaurus magnidens* [24], *Lurdusaurus arenatus* [25], and *Mantellisaurus atherfieldensis* [4,19], which possess relatively short neural spines. The remarkable elongation of their dorsal neural spines distinguishes *Morelladon*, *Hypselospinus fittoni* and *Ouranosaurus nigeriensis* from the aforementioned taxa. Nevertheless, in *Morelladon* the dorsal neural spines are vertically arranged, in contrast to the obliquely inclined neural spines of *Hypselospinus fittoni* [26]. Despite being comparatively short, the dorsal neural spines of *Barilium dawsoni* [21], *Hippodraco scutodens* [23] and *Mantellisaurus atherfieldensis* [4,19] are also posteriorly inclined. The neural spines of *Morelladon* resemble those of *Ouranosaurus nigeriensis* [27] in being more anteroposteriorly expanded distally than proximally. However the neural spines of *Ouranosaurus nigeriensis* are characterized by their extremely height (as much as 9 times centrum height). In *Morelladon* the neural spines height is more than 4.3 times the height of its respective centra. This ratio is similar to that of GPIT 1802/1 (4.5), fifth or sixth dorsal, in which the neural spine is complete in height [28]. In *Mantellisaurus atherfieldensis* the height of the spine is 2.5 times the centrum height [4], 2.8 in *Hypselospinus* (NHMUK R604), 2 in *Iguanodon bernissartensis* [4] and *Bolong yixianensis* [22], or 1 in *Lurdusaurus arenatus* (MNHN GDF 1700). Based on comparisons with *Ouranosaurus nigeriensis* [27] in which the mid-dorsal neural spines are the highest of the series, it seems likely that the ratio between the centrum height and the neural spine height might be higher for the eleventh and the twelfth dorsals of *Morelladon*. Although they are incomplete both proximally and distally, the ratio of the preserved height and the axial length of the neural spine (measured at mid-height) is equal to 4.14 in CMP-MS-03-08, 3.85 in CMP-MS-03-03 and 4.28 CMP-MS-03-16. This ratio is relatively similar to that of GPIT 1802/1 (4.25), a dorsal vertebra for an indeterminate iguanodontian from the upper Hauterivian-lower Barremian Pinilla de los Moros Formation (Burgos, Spain) [28]. The complete neural spine of this vertebra differs from the preserved neural spines of *Morelladon* by its parallel-sided anterior and posterior margins [28]. In other styracostenarns such as *Barilium dawsoni* [21], *Iguanodon bernissartensis* [4,18], *Lanzhousaurus magnidens* [24] and *Mantellisaurus atherfieldensis* [4,19] the dorsal neural spines are also parallel-sided.

#### Dorsal ribs

The proximal ends of two dorsal ribs (Fig 8) together with several fragments of dorsal rib shafts are preserved. Both proximal fragments show similar features but are distinct in size and are probably representatives of the anterior dorsal series. A long and transversely compressed neck with subparallel margins separates the capitulum and the tuberculum. In both ribs, the capitulum is thickened, rugose and suboval in outline. The tuberculum is suboval and forms a very short and thickened articular process. In both ribs the tuberculum is located dorsal to the base of the capitulum. The preserved shaft of the rib is subtriangular in cross-section with the apex pointing anterolaterally. There is a well-marked ridge originating at the base of the tuberculum that extends distally along the anterolateral surface of the shaft (Fig 8A).

#### Sacrum

The sacrum consists of six firmly co-ossified sacral vertebrae (Fig 9). The centrum of the first preserved vertebra is similar to those of the sacrodorsal in *Mantellisaurus* [19] and it is also placed anteriorly to the anterior border of the sacral yoke. However, it bears a small posteriorly directed sacrodorsal rib that contact with the sacral yoke. This first vertebra can be regarded as a sacrodorsal, so the sacrum is composed of five true sacral vertebrae (Fig 9B and 9C). The sacrum is slightly convex dorsally along its length (Fig 9C). The centrum of the sacrodorsal vertebra is large and massive with anterior and posterior articular facets that are expanded laterally and compressed dorsoventrally. The profile of the anterior facet of the centrum is elliptical and slightly indented dorsally for the neural canal. The margins of the anterior articular facet are rugose, thickened and everted. The anterior articular facet is flat showing a slight central convexity. The ventral surface of this sacrodorsal centrum is flat and subquadrangular (Fig 9B). Dorsally, the neural arch is located in the middle of the centrum. The prezygapophyses are large, ventromedially inclined and slightly overhang the anterior facet margins (Fig 9A). The short and horizontal transverse processes are located immediately posterior to the prezygapophyses, and are triangular in section with the apex facing ventrally. The base of the incomplete neural spine is anteroposteriorly short. The dorsosacral rib is borne on the ventral surface of the transverse process and the lateral surface of the neural arch. The shaft of the dorsosacral rib is thin anteroposteriorly. The distal end of the sacrodorsal rib is fused to the sacrocostal yoke. A small and circular foramen, related to the lateral nerves [18], is located in the posterior part of the neural canal wall.

Sacrals 1 to 3 have spool-like centra with modestly keeled ventral surfaces (Fig 9B). However, the keel is restricted to the anterior half of the centra in sacral vertebrae 2 and 3. In these sacral vertebrae, the neural arches and sacral ribs are clearly positioned in the intercentrum position. Because of that, the neural arch of the sacral 1 is very close to that of the sacrodorsal vertebra, what it is also evident by the closely associated facets from the sacrodorsal and first sacral ribs on the medial surfaces of the ilia. The transverse processes of the sacrals 1–3 are sub-triangular in section with the apex facing ventrally, horizontally directed, and slightly longer than those of the sacrodorsal vertebra. The bases of the neural spines of the sacrals 1–3 are fused to each other. The ribs of sacrals 1–3 are robust and ventrolaterally fused to form the sacral yoke (Fig 9B). Large foramina for lateral nerves [18] are present between the neural arches. The centra of sacrals 4 and 5 are transversely broad compared with those of sacrals 1–3. The ventral surfaces of the centra of sacrals 4–5 are subquadrangular, and sacral 4 preserves an anteroposteriorly short and transversely broad modest keel located anteriorly. In contrast, in sacral 5 the ventral surface is un-keeled and gently convex (Fig 9B). As in foregoing sacrals (s1–s3), the neural arch of sacral 4 is positioned in the intercentrum position. In sacral 5, the posterior facet of the centrum is elliptical, bears slightly expanded margins, and its surface is concave. The neural arch is positioned in the middle of the centrum. The transverse processes are short, whereas the sacral ribs are well-developed to form a large anteroventrally inclined platform in lateral view. Both postzygapophyses are preserved and they slightly overhang the posterior centrum facet margins. The postzygapophyses are small, almost vertical and have oval articular facets.

As in *Ouranosaurus* [27], the sacrum of *Morelladon* is characterized by the presence of six co-ossified sacral vertebrae. In *Barilium dawsoni* [21] and *Mantellisaurus atherfieldensis* [4,19] the sacrum comprises seven co-ossified sacral vertebrae, a condition also probably present in *Hypselospinus fittoni* (NHMUK R811) [6] and *Lurdusaurus arenatus* [25]. *Iguanodon bernissartensis* [4,18] can be distinguished from the aforementioned taxa by the presence of eight co-ossified sacral vertebrae [4]. The sacrum of *Morelladon* differs from that of *Ouranosaurus* by having a sacrodorsal with a flat ventral surface and modestly keeled ventral surfaces in the first to fourth true sacral vertebrae. As in *Ouranosaurus* the ventral surface of the last sacral vertebra is transversely convex. The sacrum of *Morelladon* can be distinguished from that of *Barilium dawsoni* [21] and *Mantellisaurus atherfieldensis* (NHMUK R5764) [19] by the absence of a midline keel in the ventral surface in the last sacral vertebral centrum. It can be also distinguished from that of *Iguanodon bernissartensis* (IRSNB 1722) [18] by the absence of a flattened ventral surface in the last sacral vertebral centrum. As in *Hypselospinus fittoni* (NHMUK R811) [6] the centrum of the first three true sacral vertebrae (s1 to s3) are spool-like and modestly keeled ventrally. However in *Hypselospinus fittoni* the centra are mildly centrally constricted [6] whereas in *Morelladon* the centra of the equivalent vertebrae are more markedly constricted (Fig 9B) and the keels of sacral 2 and 3 are restricted to the anterior half of the centra. In *Mantellisaurus atherfieldensis*, centra of sacrals 2 and 3 are markedly constricted at midlength as is seen in *Morelladon* but keeled along the entire centrum length (NHMUK R11521).

#### Haemal arches

Two haemal arches are preserved: a nearly complete one and the proximal portion of a second one (Fig 10). The proximal surface of the haemal arch is transversely expanded and bears two articular facets. In addition, a shallow ridge divides each of these articular facets into an anterior and a posterior oblique portion. The anterior portion of each articular facet is larger and more steeply inclined than the posterior portion of the facet (Fig 10B and 10E). A dorsoventrally elongate and large haemal canal is present (Fig 10A and 10D). Just below the haemal canal the shaft of the haemal arch is subcircular in section. Anteriorly along the midline of the proximal portion of the haemal arch shaft there is a shallow groove. Most of the shaft is preserved in CMP-MS-03-14 and it is thin, tubular, and curved posteriorly (Fig 10A–10C). The shaft of this haemal arch lacks its distal end and is crushed.

The fused articular facets and the morphology of the shaft, in the case of CMP-MS-03-14, suggest that both haemal arches may have been associated with anterior caudal vertebrae [21]. In addition, the thin and pointed shaft of CMP-MS-03-14 probably corresponds to the first haemal arch of the caudal series. The most complete haemal arch preserved is similar in morphology to the first haemal arch of *Mantellisaurus atherfieldensis* (IRSNB 1551) [19] and the first three haemal arches of *Iguanodon bernissartensis* (IRSNB 1534) [18].

#### Ilium

Both ilia are preserved and almost complete. The left ilium lacks the anterior end of the preacetabular process and a small area from the dorsal margin above the ischiadic peduncle (Figs 11, 12A, 12C and 12E). The right ilium is more complete but only a portion of the preacetabular process is preserved (Fig 12B, 12D and 12F). The preserved preacetabular process of the ilium is anteroposteriorly long and oriented vertically. Its curves anteroventrally and slightly laterally and becomes increasingly transversely compressed anteriorly (Fig 12A, 12C and 12E). The dorsal margin of the preacetabular process is transversely thick and rounded whereas the ventral margin is also transversely rounded but thinner. The medial surface of the preacetabular process has a prominent ridge located near its dorsal margin and extends anteriorly from the anterior border of the sacrodorsal vertebra rib facet (Fig 12C). The ventral margin of the preacetabular process curves ventrally to merge with the anterodorsal margin of the pubic peduncle (Fig 12C and 12D). The pubic peduncle is an anteroventrally extending process with a triangular cross-section. The posteroventral surface of the pubic peduncle is concave, transversely thickened and it forms the anterodorsal part of the acetabular margin. Medially, the anterodorsal margin of the pubic peduncle merges with a ridge that bordered the facet for the sacrodorsal rib and continues with the medial ridge that extends along the preacetabular process. The acetabular margin is laterally sharp and transversely broad (Fig 12E and 12F). In lateral view the acetabular margin forms a broad and deeply concave embayment (Fig 12A and 12B). The ischiadic peduncle of the ilium is strongly expanded laterally and stepped. Its anterior margin forms the posterodorsal portion of the acetabular margin. Most of the dorsal margin of the main iliac blade displays a slight lateral overhang to its dorsolateral edge. The dorsal margin of the ilium above the pubic and ischiadic peduncles and acetabulum is straight in lateral view (Fig 12B). From the base of the preacetabular process, the dorsal margin of the iliac blade thickens posteriorly to form a laterally everted rim dorsal to the ischiadic peduncle. From this point the dorsal margin of the postacetabular process of the ilium slopes posteroventrally (Fig 12A and 12B). The postacetabular process of the ilium is triangular and tapers posteriorly in lateral view (Fig 12A and 12B). Posterior to the ischiadic peduncle the ventral margin of the postacetabular process inclines posterodorsally to meet the dorsal surface at the distal end. The distal end of the postacetabular process is expanded and its ventral surface forms a shallow arched brevis fossa. Laterally a distinct ridge bounds the brevis fossa whereas its medial margin is sharp. The medial surface of the ilium possesses a pronounced ridge that extends from the preacetabular medial ridge to the posterior tip of the postacetabular process. Along this ridge the medial surface is excavated by a series of facets for attachment of the sacral transverse processes and dorsal parts of the sacral ribs (Fig 12C and 12D). Three sacral rib facets are clearly distinguishable; the first two are closely packed and located near the dorsal margin of the ilium, whereas the third one is located above the ischiadic peduncle and slightly below the previous facets (Fig 12C and 12D). Posterior to this third facet the medial surface of the postacetabular process below the ridge is striated. Immediately posterior to the ischiadic peduncle the medial ridge is upwardly directed parallel the ventral margin of the postacetabular process. This medial ridge meets the dorsal margin of the ilium level to the inflection point in which the dorsal margin slopes posteroventrally. At this point, the medial ridge also extends parallel to the dorsal margin of the iliac blade to meet the distal end, and forms a medially expanded and shallowly concave platform.

The ilium of *Morelladon* is similar in morphology to that of *Mantellisaurus atherfieldensis* (IRSNB 1551, NHMUK R5764, R6462, R11521) [19,29,30] and *Hypselospinus fittoni* (NHMUK R1635) [6]. However, *Morelladon* lacks the small medioventral flange, medial to the brevis fossa, that is visible laterally in the postacetabular process of the ilium in *Mantellisaurus atherfieldensis* (NHMUK R11521, R6462) [4,19] and *Hypselospinus fittoni* (NHMUK R1635) [6]. A similar medioventral flange to that of *Hypselospinus* is present in the ilium of *Lurdusaurus arenatus* [25]. As in *Hypselospinus fittoni* (NHMUK R1834), *Iguanacolossus fortis* [23], *Lurdusaurus arenatus* [25] and *Ouranosaurus nigeriensis* [27], the preacetabular process of the ilium in *Morelladon* is transversely compressed and oriented vertically, differing from the lateral torsion shown by *Barilium dawsoni* [21], *Bolong yixianensis* [21], *Delapparentia turolensis* [2], *Iguanodon bernissartensis* [4,18], *Iguanodon galvensis* [5], *Mantellisaurus atherfieldensis* [4,19] and *Proa valdearinnoensis* [6]. In *Morelladon* the ischiadic peduncle of the ilium is strongly expanded laterally and stepped as in *Barilium dawsoni* [21], *Hypselospinus fittoni* [6] and *Mantellisaurus atherfieldensis* [19]. The ilium of *Morelladon* can be distinguished from that of *Barilium dawsoni* [21], *Bolong yixianensis* [22], *Delapparentia turolensis* [2], *Iguanodon bernissartensis* [4,18], *Iguanodon galvensis* [5], *Ouranosaurus nigeriensis* [27] and *Proa valdearinnoensis* [3] in having a straight dorsal margin between the pubic and ischiadic peduncles. The presence of a straight dorsal margin of the iliac blade is a feature shared with *Hypselospinus fittoni* [6], *Jinzhousaurus yangi* [20], *Lurdusaurus arenatus* [25] and *Mantellisaurus atherfieldensis* [4,19]. As in the latter taxon [4] the dorsal margin of the main iliac blade of *Morelladon* presents a slightly lateral overhang to its dorsolateral margin. The ilia of *Morelladon* and *Barilium dawsoni* [21] differ in the morphology of the postacetabular process. In the former, it is triangular and tapers posteriorly, while in the latter the ventral margin lies horizontally [4]. A horizontal ventral margin of the postacetabular process is also present in *Bolong yixianensis*, but this styracosternan possesses a remarkably dorsoventrally narrow postacetabular process [22]. The ilium of *Morelladon* can be differentiated from that of *Iguanodon bernissartensis* [4,18] and *Iguanodon galvensis* [5] by the presence of an inflection point in the dorsal margin of the postacetabular process that abruptly slopes posteroventrally (Fig 12A and 12B). The absence of a laterally visible brevis shelf and fossa is a feature that distinguishes *Morelladon* from *Jinzhousaurus yangi* [20]. The presence of a medial ridge on the postacetabular process that slopes posterodorsally to meet the point in which the dorsal margin of the ilium slopes posteroventrally is a unique feature of *Morelladon* (see arrow in Fig 12E). In *Hypselospinus fittoni* (NHMUK R1635), *Iguanacolossus fortis* [23], *Lurdusaurus arenatus* [25] and *Ouranosaurus nigeriensis* [27], and *Osmakasaurus depressus* [31], the medial ridge on the postacetabular process is horizontal. As in *Morelladon*, the medial ridge of the postacetabular process of *Mantellisaurus atherfieldensis* (NHMUK R11521, R6462) [19], slopes posterodorsally but ends in the posterior tip of the postacetabular process not reaching the dorsal margin.

#### Pubis

Both pubes are very incomplete. The right pubis comprises part of the acetabular margin, the iliac peduncle and the proximal portion of the anterior process (Figs 11, 13E and 13F); whereas the left pubis preserves the iliac peduncle, the acetabular margin, part of the ischial peduncle and the base of the posterior process (Fig 13G and 13H). The preserved anterior process is dorsoventrally deep, blade-like and transversely compressed. Laterally, the dorsal margin of the anterior pubic process curves anterodorsally whereas the ventral margin is sinuous and anteriorly curves anteroventrally, so the presence of a dorsoventrally expanded distal tip cannot be ruled out (Fig 13E and 13F). The dorsal margin of the anterior process is transversely thick, rounded and curves medially, whereas the ventral margin is transversely thin and sharp-edged. The iliac peduncle is subtriangular in posterior view, and it is located posterodorsal to the base of the anterior process. The subtriangular acetabular surface is dorsoventrally concave and ventrally directed (Fig 13E and 13F). The ischial peduncle is incomplete. It is located near the base of the posterior pubic process just above the obturator foramen. Associated with the obturator foramen there is an oblique obturator channel located at the base of the posterior pubic process. Just the proximal portion of the posterior pubic process is preserved (Fig 13G and 13H) being rod-shaped and roughly circular in cross-section.

The morphology of the preserved pubes of *Morelladon* is similar to the equivalent portion in *Barilium dawsoni* (NHMUK R802) [21], *Delapparentia turolensis* [2], *Hypselospinus* (NHMUK R811) [6], *Iguanodon bernissartensis* (IRSNB 1534) [18] *Mantellisaurus atherfieldensis* (IRSNB 1551, NHMUK R5764) [19,26] and *Ouranosaurus nigeriensis* [27]. Despite the anterior pubic process lacking its anterior end, it seems likely that the dorsoventral expansion of the anterior pubic process of *Morelladon* begins at the midpoint of the length of the process as in *Hypselospinus* (NHMUK R1831) [6], *Mantellisaurus atherfieldensis* (IRSNB 1551, NHMUK R5764) [19,26] and *Ouranosaurus nigeriensis* [27]. *Morelladon* can be distinguished from *Delapparentia turolensis* [2], *Lurdusaurus arenatus* [32], *Iguanodon bernissartensis* (IRSNB 1534) [18], *Iguanodon galvensis* [5] and *Proa valdearinnoensis* [3] by the presence of an anterior pubic process that is short and deep. This condition is similar to that of *Hypselospinus* (NHMUK R1831) [6], *Lanzhousaurus magnidens* [28], *Mantellisaurus atherfieldensis* (IRSNB 1551, NHMUK R5764) [19,26] and *Ouranosaurus nigeriensis* [27]. This contrasts with the relatively elongate and narrow anterior pubic process of *Barilium* (NHMUK R3788) [21], *Delapparentia turolensis* [2], *Iguanodon bernissartensis* (IRSNB 1534) [18], *Iguanodon galvensis* [5], *Jinzhousaurus yangi* [20] and *Proa valdearinnoensis* [3]. As in *Hypselospinus* [6], *Iguanodon* [18], *Mantellisaurus* [19], *Jinzhousaurus* [20], *Ouranosaurus* [27] and *Proa* [3] the rod-shaped posterior process of the pubis in *Morelladon* is circular.

#### Ischium

The left ischium is nearly complete (Figs 11, 13A and 13B), but only the ischial peduncle and two fragments of the ischial shaft were recovered from the right ischium (Fig 13C and 13D). The proximal end of the ischium is anteroposteriorly expanded and transversely compressed. This proximal end is divided into two processes, the anterodorsal pubic and the posterodorsal iliac peduncles (Fig 13A and 13B). The articular surface of the pubic peduncle is rugose and triangular. The posterior margin of the pubic peduncle forms the ventral portion of the acetabulum. The ventral margin of the pubic peduncle is not completely preserved, but in lateral view, the pubic peduncle is narrower than the iliac peduncle. The articular surface of the iliac peduncle is sub-rectangular, highly rugose and bears a central depression. The anterior margin of the iliac peduncle forms the posterodorsal part of the acetabular margin. In lateral view, the posterior margin of the iliac peduncle displays a pronounced curvature (Fig 13A and 13D). In lateral view the acetabular margin is markedly concave. Laterally, the acetabular margin possesses a clearly defined stepped groove that extends along it between the iliac and pubic peduncles (Fig 13A). The obturator process is located anteriorly on the ventral margin of the ischial shaft. The obturator process is an anteriorly projecting and large sub-triangular process that ends in a rounded tip and is transversely compressed (Fig 13A and 13B). The elongated shaft of the ischium is probably straight along its length (Fig 13A and 13B). Proximally, the shaft is sub-triangular in section, with the apex facing laterally, and bears a prominent ridge that extends longitudinally from the base of the pubic peduncle to midshaft in lateral view. A second prominent lateral ridge extends longitudinally from the base of the obturator process to midshaft. From midlength, the section of the ischial shaft becomes ellipsoidal, grading to semicircular distally, with a convex lateral surface and a flat medial surface. In addition, the medial surface of the ischial shaft immediately posterior to midlength is highly striated, representing the ischial symphysis. The distal end is slightly anteroposteriorly and laterally expanded but does not form an expanded boot (Fig 13A and 13B). The distal surface is rugose and sub-triangular in ventral view.

The morphology of the ischium of *Morelladon* is similar to those of *Mantellisaurus* [19] and *Jinzhousaurus* [20]. As in *Mantellisaurus* [19] the shaft of the ischium is narrow and angular-sided. The ischium of *Morelladon*, *Jinzhousaurus* and *Mantellisaurus* can be distinguished from those of *Delapparentia* (MPZ 2014/328) [33], *Hypselospinus* (NHMUK R811) [6], *Iguanodon bernissartensis* [23] and *Lurdusaurus arenatus* [32] in having a straight ischial shaft. In *Ouranosaurus* the ischial shaft is slightly bowed dorsally at its distal portion [27], whereas in *Bolong* the preserved shaft of the left ischium is bowed, but it is straight in the right ischium [22]. A markedly concave acetabular margin is present in the ischia of *Morelladon*, *Hippodraco* [23], *Iguanodon bernissartensis* [18], *Mantellisaurus* [19] and *Ouranosaurus* [27], differing from the gently concave acetabular margin of *Barilium* (NHMUK R802) [21], *Bolong* [22], *Delapparentia* [2,33], *Hypselospinus* (NHMUK R1635, R811) [6] and *Jinzhousaurus* [19]. Both in *Bolong* [22], *Lurdusaurus* [25] and *Ouranosaurus* [27], the obturator process is closer to the pubic peduncle than in *Barilium* (NHMUK R3788) [21], *Morelladon* (Fig 13A and 13B), *Mantellisaurus* (NHMUK R11521), *Hypselospinus* (NHMUK R1635, R811) [6] and *Iguanodon bernissartensis* (IRSNB 1534) [18], enclosing a deep and narrow embayment. In the latter taxa, this embayment is wider and less deep. The ischium of *Morelladon* differs from *Mantellisaurus* (NHMUK R11521) [4] and *Jinzhousaurus* [20] in lacking an anteriorly expanded boot in its distal end. An expanded boot is also present in *Hypselospinus* (NHMUK R811) [6], *Lurdusaurus arenatus* [32], *Iguanodon* [18] and *Ouranosaurus* [27]. In *Bolong yixianensis* the distal end of the ischium terminates in a moderately expanded knob [22]. In *Ouranosaurus* [27] the pubic peduncle is wider than the iliac peduncle, whereas in *Morelladon* and other European basal stryracosternans such as *Delapparentia* [33], *Iguanodon bernissartensis* (IRSNB 1534) [18], *Hypselospinus* (NHMUK R811) or *Mantellisaurus* (NHMUK R11521), the reverse is the case. As in *Hypselospinus* (NHMUK R1635) and *Mantellisaurus* (NHMUK R11521) a lateral stepped groove is present immediatedly below the acetabular margin between the iliac and pubic peduncles.

#### Tibia

Only the left tibia is preserved and is nearly complete (Fig 14). The proximal end of the tibia is expanded anteroposteriorly (Fig 14A and 14B). The flat proximal surface is highly rugose and inclined posteroventrally. The blade-shaped cnemial crest is compressed transversely and slightly curves laterally (Fig 14D). The anterior margin of the cnemial crest is rounded in lateral or medial view (Fig 14A and 14B). The medial surface is convex whereas the lateral surface is gently concave. The fibular condyle projects laterally and possesses a shallow notch in the midposterior part. The posterior margin of the tibia and the fibular condyle are separated by a deep and narrow cleft (Fig 14E). The shaft of the tibia is straight.

The distal end of the tibia is expanded transversely and is distinctly asymmetrical (Fig 14A and 14B). The lateral and medial malleoli are separated by a pronounced step (Fig 14A). The lateral malleolus projects further ventrally than the medial malleolus. The flat anterior surface of the lateral malleolus, with which the fibula articulates, is striated. The lateral margin of the lateral malleolus is rounded, narrow and slightly expands distally. The medial malleolus is triangular and projects farther anteromedially (Fig 14F). The medial surface of the medial malleolus is flat whereas the anterior surface is convex. Distally the facet for the astragalus is rugose.

The tibia of *Morelladon* is very similar in morphology to those of *Mantellisaurus* [18] and *Ouranosaurus* [27] and more slender and lightly built than in *Iguanodon bernissartensis* [18]. The tibia of *Ouranosaurus* differs from *Morelladon* in having a ventrally inclined cnemial crest in lateral view [27], whereas in *Lurdusaurus* the cnemial crest is anterodorsally inclined [25]. The tibia of *Morelladon* can be distinguished from that of *Barilium dawsoni* (NHMUK R4771) [21] and *Mantellisaurus atherfieldensis* (IRSNB 1551) [19] by the absence of a prominent cnemial crest. Differing from these two species, ventral to the proximal expansion, the tibia of *Morelladon* gently narrows to form the shaft (Fig 14A and 14B). However, the cnemial crest of the tibia of *Morelladon* is not as weakly developed as in *Bolong yixianensis* (YHZ-001) [22].

### Phylogenetic analysis

**Fig 15:**
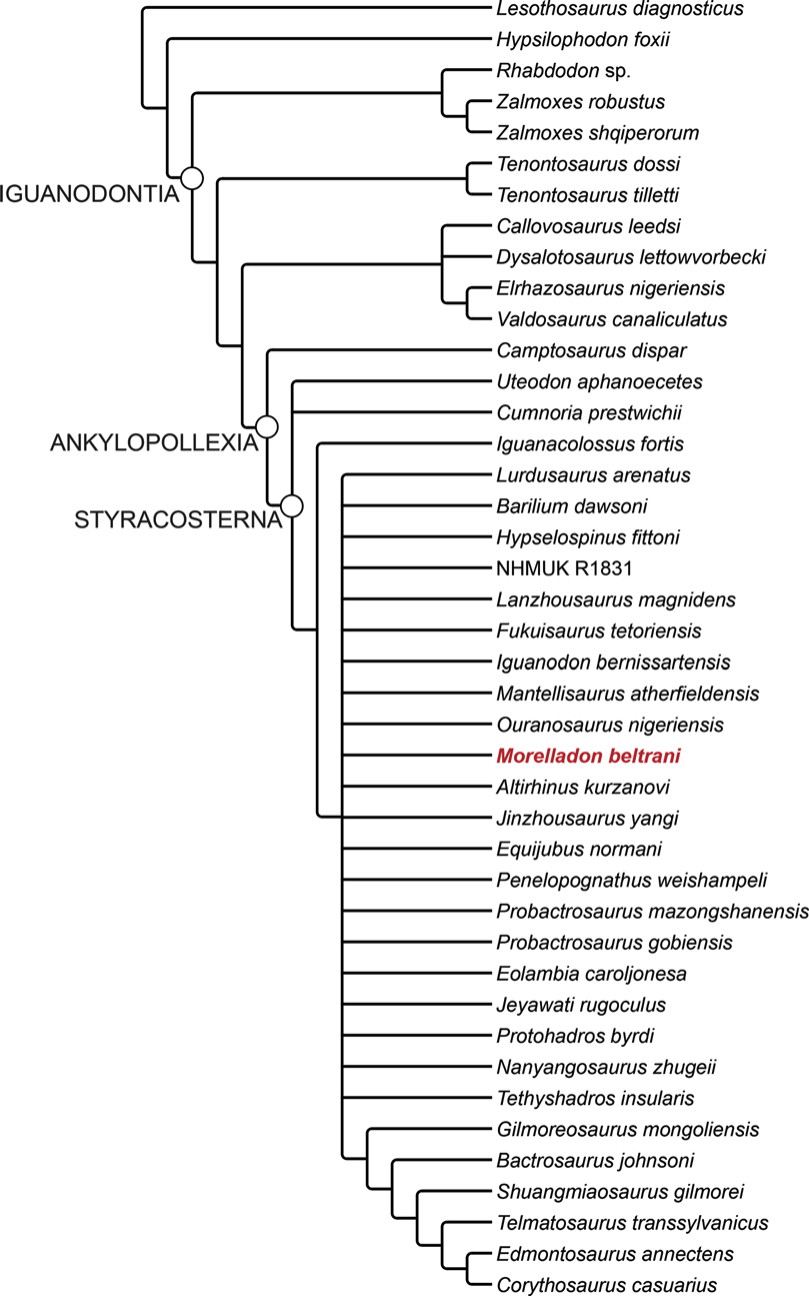
Phylogenetic relationships of *Morelladon beltrani* (CMP-MS-03). Strict reduced consensus subtree of 801 most parsimonious trees resulting from a second analysis, after deletion of 27 taxa (see text for details), of the modified data matrix from McDonald et al. [3].

To establish the phylogenetic relationships of *Morelladon* we carried out two separate phylogenetic analyses. A first analysis was based on the data matrix of McDonald et al. [3], which was recently modified to include new data from *Fukuisaurus* and *Koshisaurus* [34] and *Delapparentia* [35]. In addition, as in Shibata and Azuma [34], *Kukufeldia* was excluded whereas character 110 was scored following Gasca et al. [35]. The final data matrix includes 69 taxa and 135 characters (S2 Supporting Information) and was processed with the phylogenetic software TNT [36] using a ‘Traditional search’ (the starting trees were Wagner with a random seed of 1 and 9999 replicates; the tree bisection reconnection algorithm was used with 10 trees saved per replication). The analysis recovered 3240 most parsimonious trees (MPTs) with lengths of 427 steps [Consistency Index (CI) = 0.468, Retention Index (RI) = 0.797]. As in McDonald et al. [3], the strict consensus tree was very poorly resolved. Two iguanodontian clades are supported: Rhabdodontidae (*Muttaburrasaurus*, *Rhabdodon*, (*Zalmoxes robustus*, *Z*. *shqiperorum*)) and *Tenontosaurus* (*T*. *dossi* + *T*. *tilletti*), but nearly the whole of Iguanodontia is recovered in an unresolved polytomy. In order to improve the resolution a second search was made. Using PAUP 4.10b [37], the maximum agreement subtree was calculated, but *Morelladon* was among the 27 taxa excluded from the subtree. To better explore the systematic position of *Morelladon*, a new data matrix was composed removing all the taxa excluded in the maximum agreement subtree, but not *Morelladon*. This new data matrix included 42 taxa and 135 characters and was analysed in TNT with the same procedure described above. This analysis recovered 801 maximum agreement subtrees with lengths of 347 steps, [Consistency Index (CI) = 0.576, Retention Index (RI) = 0.869]. In the strict reduced consensus tree (Fig 15), in addition to Iguanodontia, Ankylopollexia and Stryracosterna were also recovered as clades. Nevertheless, with the exception of *Iguanacolossus* and the clade (*Gilmoreosaurus* (*Bactrosaurus*, (*Shuangmiaosaurus* (*Telmatosaurus*, (*Edmontosaurus*, *Corythosaurus*))))), the rest of styracosternans, including *Morelladon*, form an unresolved polytomy.

**Fig 16:**
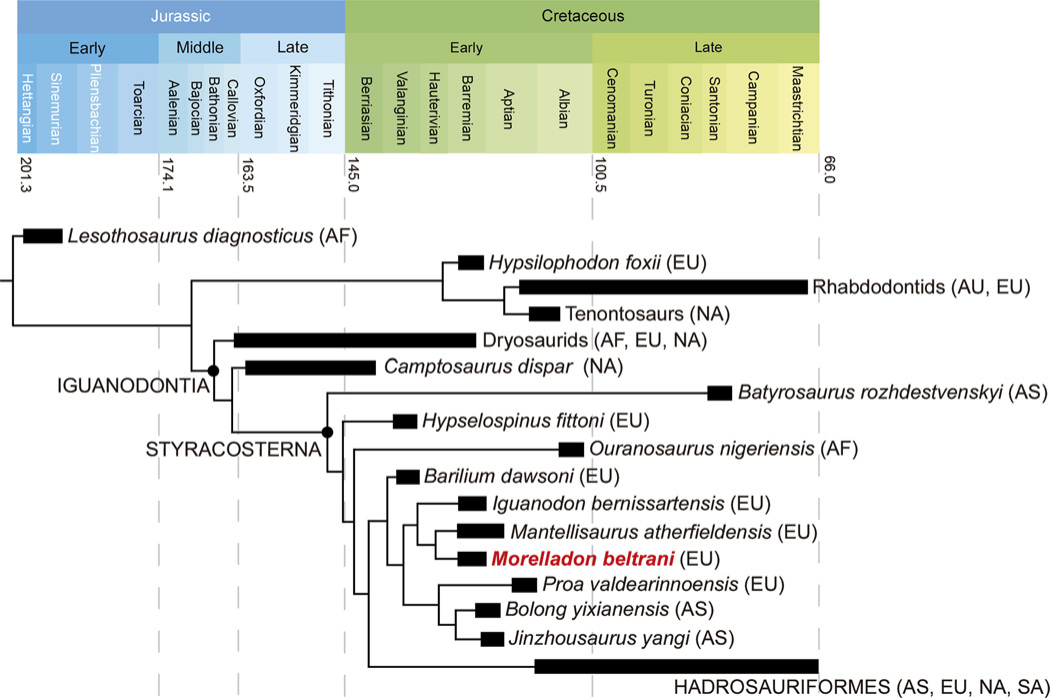
Phylogenetic relationships of *Morelladon beltrani* (CMP-MS-03). Time-calibrated strict consensus tree resulting from the analysis of the modified data matrix from Norman [6]. Abbreviations: AF, Africa; AS, Asia; AU, Australia; EU, Europe; NA, North America, SA, South America.

A second analysis was performed using the data matrix of Norman [6]. The data matrix included 28 taxa and 105 characters (S3 Supporting Information) and was analyzed using TNT [36] using a ‘Traditional search’. The starting trees were Wagner with a random seed of 1 and 1000 replicates: the tree bisection reconnection algorithm was used with 100 trees saved per replication. The analysis of the matrix of Norman [6] recovered just one most parsimonious tree (MPT) with lengths of 314 steps [Consistency Index (CI) = 0.576, Retention Index (RI) = 0.781]. Note that character state 1 for character 72 (middle to posterior dorsal vertebra neural spine proportions) has been reinterpreted and recoded for tall and narrow, axial length <50% of height. The tree (Fig 16) recovered a similar topology to that obtained by Norman [6] and places *Morelladon* as the sister taxon to *Mantellisaurus atherfieldensis* in a clade with the topology (*Iguanodon bernissartensis*, (*Morelladon*, *Mantellisaurus*)) within non-hadrosauriform Styracosterna. *Morelladon* and *Mantellisaurus* are united by sharing a straight (character 95, state 0), narrow and angular-sided (character 96, state 2) ischial shaft. As in the tree topology of Norman [6] ‘iguanodontoid’ styracosternans are recovered as the sister-clade of Hadrosauriformes (*sensu* Norman [6]). However, the tree topology obtained here differs from that reported by Norman [6] in the position of *Barilium dawsoni*. *Barilium dawsoni* is here recovered as the most basal ‘iguanodontoid’ differing from the position recovered in the strict consensus tree of Norman [6] which places *Barilium* within an unresolved polytomy at the base of ‘iguanodontoid’ styracosternans that included a clade consisting of *Iguanodon bernissartensis* and *Mantellisaurus* and a clade with the topology (*Proa*, (*Jinzhousaurus*, *Bolong*)). Other trees derived for the analysis of Norman [6] place *Barilium dawsoni* as the basal taxon of a clade with the topology (*Barilium*, (*Iguanodon bernissartensis*, *Mantellisaurus*)) (Figs. 50–52 in [6]). In all of these hypotheses, *Barilium* is a representative of the sister-clade of (*Proa*, (*Jinzhousaurus*, *Bolong*)) clade within the ‘iguanodontoid’ styracosternans.

Despite the differences in the character and taxon sampling of the data matrices, both analyses place *Morelladon* close to the Barremian**-**lower Aptian European strycosternans *Iguanodon bernissartensis* and *Mantellisaurus atherfieldensis*, also known from the Mas de la Parreta quarry in Spain [9].

## Conclusions

Despite the limited material known from the type specimen, a suite of autapomorphies supports the validity of *Morelladon beltrani*. In addition, *Morelladon* can be distinguished from other styracosternan iguanodontians on the basis of a unique combination of characters. Regardless of the phylogenetic analysis carried out, *Morelladon* is clearly nested within the clade that contains its synchronic and sympatric contemporary European taxa *Iguanodon bernissartensis* and *Mantellisaurus atherfieldensis*, and the lower Albian *Proa valdearinnoensis* from the Iberian Peninsula.

Styracosternans are by far the most abundantly represented group of dinosaurs in the Arcillas de Morella Formation including, besides *Morelladon beltrani*, several individuals assigned to *Iguanodon bernissartensis* and probably to *Mantellisaurus atherfieldensis*. In addition, the recognition of *Morelladon beltrani* provides a distinguishing component of the Arcillas de Morella Formation respect to its equivalent upper Wealden facies from northwest Europe (Belgium and England), also characterized by the presence of the medium-large bodied styracosternans *Iguanodon bernissartensis* and *Mantellisaurus atherfieldensis*. Finally, the discovery of *Morelladon beltrani* gen. et sp. nov. combined with other recent named taxa (e.g., *Delapparentia turolensis* [2], *Proa valdearinnoensis* [3], *Iguanodon galvensis* [5]) recognizes the Iberian Peninsula as a highly diverse Early Cretaceous medium-large bodied styracosternan assemblage worldwide.

## Supporting Information

S1 Supporting Information Tables of measurements. (PDF)

S2 Supporting Information Data Matrix 1. (TNT)

S3 Supporting Information Data Matrix 2. (TNT)
