# Supplementary material for: A New Sail-Backed Styracosternan (Dinosauria: Ornithopoda) from the Early Cretaceous of Morella, Spain
Source: PLoS One. 2015 Dec 16;10(12):e0144167. doi: 10.1371/journal.pone.0144167 (PMC4691198; doi:10.1371/journal.pone.0144167)
Supplement: S1 Supporting Information — (PDF) [file pone.0144167.s001.pdf]

S3. Supporting Information

Table 1. Measurements (all given in millimetres) of the dorsal vertebrae and neural spines.

	CL	AFW	AFH	PFW	PFH	NH	NLb	NLd
**CMP-MS-03-10**	70	63.5	63.5	69.5	67		64	
**CMP-MS-03-09**	93.5	72	62.5	75	64.5		68	
**CMP-MS-03-06**	93	59	79	67	80		76	
**CMP-MS-03-05**	93	69.5	76*	74	75*		65*	
**CMP-MS-03-07**	89	66	81	74	82		78*	
**CMP-MS-03-04**	78	82	91	87	96		64*	
**CMP-MS-03-03**	81	82*	58*	101	69*			
**CMP-MS-03-18**						165*	64*	65*
**CMP-MS-03-016**						240*	54*	63.5*
**CMP-MS-03-19**						215*	60*	69*
**CMP-MS-03-08**						287*	63*	81*
**CMP-MS-03-17/29**						320*	65*	81*
**CMP-MS-03-15**						237*	76*	84*
**CMP-MS-03-03**						210*	61*	60*

CL Centrum length; AFW Anterior facet width; AFH Anterior facet height; PFW Posterior facet width; PFH Posterior facet height; NH Neural spine height; NLb Neural spine length (measured at base); NLd Neural spine length (measured distally)

*Measurement of the preserved element (not complete).

Table 2. Measurements (all given in millimetres) of the pelvic elements and left tibia.

Element	Measurement	Dimension (mm)
Left ilium	Greatest length	555*
Left ilium	Height over the acetabulum	142
Left ilium	Height over ischiadic peduncle	198
Left ilium	Greatest length of ischiadic peduncle measured transversely	64
Left ilium	Postacetabular process length	186
Right ilium	Greatest length	525*
Right ilium	Height over the acetabulum	148
Right ilium	Height over ischiadic peduncle	201
Right ilium	Greatest length of ischiadic peduncle measured transversely	55
Right ilium	Postacetabular process length	191
Left pubis	Greatest length of prepubic process measured dorsoventrally	96
Left ischium	Estimated dorsoventral length measured from anterior border of the iliac peduncle to the distal end	773
Left ischium	Estimated dorsoventral length measured from posterior border of the iliac peduncle to the distal end	738
Left ischium	Estimated dorsoventral length measured from the pubic peduncle to the distal end	758
Left ischium	Greatest length of distal end measured dorsoventrally	75
Left ischium	Greatest length of distal end measured transversely	47
Right ischium	Greatest length of iliac peduncle measured anteroposteriorly	112
Left tibia	Greatest length of proximal end measured anteroposteriorly	225
Left tibia	Greatest length of proximal end measured transversely	103
Left tibia	Greatest length of distal end measured anteroposteriorly	111
Left tibia	Greatest length of distal end measured transversely	188

*Measurement of the preserved element (not including the distal fragments of the preacetabular processes on both ilia).
